# Stem Cell‐based therapies for COVID‐19‐related acute respiratory distress syndrome

**DOI:** 10.1111/jcmm.17265

**Published:** 2022-04-14

**Authors:** Hoi Wa Ngai, Dae Hong Kim, Mohamed Hammad, Margarita Gutova, Karen Aboody, Christopher D. Cox

**Affiliations:** ^1^ Department of Stem Cell Biology and Regenerative Medicine City of Hope Beckman Research Institute Duarte California USA; ^2^ StemXO Inc Long Beach California USA

**Keywords:** COVID‐19, mesenchymal stem cells, stem cells, therapeutics

## Abstract

As the number of confirmed cases and resulting death toll of the COVID‐19 pandemic continue to increase around the globe ‐ especially with the emergence of new mutations of the SARS‐CoV‐2 virus in addition to the known alpha, beta, gamma, delta and omicron variants ‐ tremendous efforts continue to be dedicated to the development of interventive therapeutics to mitigate infective symptoms or post‐viral sequelae in individuals for which vaccines are not accessible, viable or effective in the prevention of illness. Many of these investigations aim to target the associated acute respiratory distress syndrome, or ARDS, which induces damage to lung epithelia and other physiologic systems and is associated with progression in severe cases. Recently, stem cell‐based therapies have demonstrated preliminary efficacy against ARDS based on a number of preclinical and preliminary human safety studies, and based on promising outcomes are now being evaluated in phase II clinical trials for ARDS. A number of candidate stem cell therapies have been found to exhibit low immunogenicity, coupled with inherent tropism to injury sites. In recent studies, these have demonstrated the ability to modulate suppression of pro‐inflammatory cytokine signals such as those characterizing COVID‐19‐associated ARDS. Present translational studies are aiming to optimize the safety, efficacy and delivery to fully validate stem cell‐based strategies targeting COVID‐19 associated ARDS for viable clinical application.

## 1 COVID‐19, ACUTE RESPIRATORY DISTRESS SYNDROME, AND MULTI‐ORGAN INVOLVEMENT

The novel 2019 coronavirus disease (COVID‐19) was first identified in Wuhan, China with the emergence of a cluster of pneumonia cases in December 2019 and was declared a pandemic by the World Health Organization (WHO) on 11 March 2020.^1^ As of 30 December 2021, there have been 53,795,407 confirmed cases and 820,355 deaths in the United States alone according to the Centers for Disease Control and Prevention, with a global total of 290,959,019 cases and 5,446,753 deaths according to the World Health Organization as of 4 January 2022.^2^ The causative pathogen for COVID‐19 is the severe acute respiratory syndrome coronavirus‐2 (SARS‐CoV‐2), a distinct strain of coronavirus related to those that resulted in the severe acute respiratory syndrome (SARS) pandemic in 2003 and the Middle East respiratory syndrome (MERS) pandemic in 2012.^3^ Because of its recent emergence, much remains to be elucidated regarding the pathophysiological mechanisms, sequelae and strength and duration of the host immune response in SARS‐CoV‐2, despite a tremendous amount of research worldwide. SARS‐CoV‐2 has demonstrated high genetic variability and a rapid mutation rate, and preliminary evidence suggests that immune protection may be limited, providing challenges to the development of vaccines and treatments.^4^ The persistent emergence of novel SARS‐Cov‐2 variants, highlighted by the Delta and Omicron examples, present continued concerns that novel strains capable of effective vaccine escape and/or heightened virulence will emerge, rendering present vaccines ineffective in preventing infection. Even where vaccines are effective, meeting global demands to provide vaccine accessibility to local populations has proven challenging. The emergence of a contradictory but sizeable body of evidence that efficacy of present vaccines against SARS‐Cov‐2 wanes after 4–6 months further complicates meeting this demand.^5^, ^6^ Collectively, these issues underscore the clear need that persists for the development of effective therapies to address SARS‐Cov‐2 infection and its more severe clinical presentations.

COVID‐19 is characterized by a diverse number of potential complications, both respiratory and non‐respiratory. A recent study conducted in Wuhan, China examining 201 hospitalized COVID‐19 pneumonia patients for example, demonstrated that 41.8% of the patients developed acute respiratory distress syndrome (ARDS) during COVID‐19‐related pneumonia, with the subset of patients progressing to ARDS exhibiting a mortality rate of 52.4%.^7^ With a median patient age of 51, this case study also underscored the elevated susceptibility of older subpopulation to ARDS and consequently a substantially higher risk of mortality. ARDS is a life‐threatening lung pathology characterized by rapid onset and resulting from a massive and generalized pro‐inflammatory immune response in the lungs, circulation and other tissues in COVID‐19 patients; this cytokine storm represents the most life‐threatening development in COVID‐19 patients.^8^ In non‐COVID patients, ARDS typically arises as a complication of pneumonia, systemic infection and major trauma,^9^ and it is associated with elevated transport of fluid from lung capillaries to alveoli,^10^ the air sacs that are the site of gas exchange with the blood, resulting in pulmonary oedema, hypoxemia, and loss of lung compliance secondary to epithelial damage and pulmonary fibrosis.

The cytokine storm characterizing COVID‐19 ARDS has also been implicated in tissue damage and embolus formation in multiple organ systems and to play a key role in the pathophysiology of extrapulmonary multiple organ failure secondary to ARDS^11^, ^12^; indeed, this process is hypothesized to be key in the development of a number of emergent chronic post‐COVID‐19 pathologies. For instance, emerging evidence investigating the development of ‘post‐viral syndrome’ in a subset of post‐recovery COVID‐19 patients is examining possible corollaries with earlier SARS variants which produced a chronic pathological state resembling chronic fatigue/myalgic encephalomyelitis as a result of viral infiltration to select brain regions.^13^, ^14^ Histopathological examination of brain tissue in necropsied patients has also demonstrated neurological complications in a subset of COVID‐19 patients implicating non‐inflammatory neurovascular damage in clinical manifestations ranging from loss of olfaction/gustation to loss of involuntary control of breathing through medullary centres, with the virus hypothesized to spread to the brain from the upper respiratory tract via the transcribrial route, where angiotensin‐converting enzyme 2 (ACE2)‐expressing tissues enable viral internalization.^15^ Evidence from other coronaviruses in human case reports and in vivo models also suggest the possibility of brain lesioning and fatal encephalitis.^16^, ^17^, ^18^, ^19^, ^20^, ^21^

## 2 OVERVIEW OF COVID‐19 ARDS PATHOPHYSIOLOGY

SARS‐Cov‐2 is a large, enveloped Betacoronavirus of the Coronaviridae family, order Nidovirales, which it shares with 5 other species and 6 other total strains of the coronavirus family known to infect humans to date. These include the Alphacoronaviruses HCov‐229E and HCov‐NL63; and the Betacoronaviruses HCov‐OC43, HCov‐HKU1, SARS‐Cov (now often designated SARS‐Cov‐1 to avoid confusion), and MERS‐Cov.^22^ SARS‐Cov‐1, with which SARS‐Cov‐2 exhibits 77.5% sequence homology,^23^ was causative of the 2002–2004 SARS outbreak, characterized by variable virulence with localized mortality rates as high as 17%^24^ and was noted for mutating to optimize viral‐host binding and replication^25^; the Middle East respiratory syndrome (MERS‐Cov) virus, with which SARS‐Cov‐2 shares 50% sequence homology^23^ and which enters host cells via the DPP4 receptor, exhibits a mortality rate of 35%.^26^, ^27^ Symptoms of the other human coronavirus strains are generally mild with low rates of mortality and morbidity and these infections are typically associated with the ‘common cold’, accounting for 10%–30% of all adult upper respiratory infections annually.^22^

Coronaviruses are comprised of single‐stranded, positive‐sense RNA genomes of 28–32 kb in length, the largest genomes of all RNA viruses, and these are translated to non‐structural proteins through two open‐reading frames.^28^ SARS‐Cov‐2 displays a surface Spike S glycoprotein on its viral envelope that is critical for host receptor binding^29^ and invades host cells via interaction with the angiotensin‐converting enzyme 2 (ACE2) receptor and is subsequently internalized where it integrates within the host genome and exploits its machinery for viral replication. ACE2 receptors are widely distributed in lung alveolar epithelia as well as nasopharyngeal and oral mucosa cells; extra‐pulmonary expression is widely distributed among tissues spanning multiple physiological systems, however, including the liver, kidneys, gut, endothelial and vascular smooth muscle, and brain, providing a mechanism for multi‐organ involvement.^30^

## 3 STEM/PROGENITOR CELL THERAPIES FOR THE TREATMENT OF ARDS

### 3.1 Candidates for cell‐based therapies

Despite years of efforts by multiple investigative teams aiming to develop viable treatments for ARDS, many candidate therapies have failed to show efficacy.^31^ While corticosteroidal anti‐inflammatory drugs such as dexamethasone and hydrocortisone^32^, ^33^, ^34^ or interleukin receptor antagonists^35^, ^36^, ^37^, ^38^, ^39^ have been investigated for ARDS and COVID‐associated ARDS with evidence of success for severely ill/ventilated patient outcomes in a large number of instances, study outcomes to date have also proven inconsistent with these interventions, with steroidal interventions in some cases even elevating mortality in related cases of ARDS with influenza, leading to hesitancy among many clinicians to rely on these—especially given the lack of clarity on risk factors in instances of contrainidication.^33^ In the case of broad immuno‐suppressant effects with cytokine‐suppressant drugs for instance, it has been hypothesized that immunosuppression may worsen infection which in some cases outweighs the beneficial effects of cytokine storm suppression, depending on the stage of disease and pre‐existing immune status of the patient.

More recently, certain stem cell‐based therapies are beginning to show promising results in mitigating ARDS symptoms. Key examples of these candidate interventions are shown in Table 1. These carry a number of predicted advantages over corticosteroidal or receptor antagonist‐based pharmacological interventions, including their intrinsic inflammatory‐suppressant properties which combine with a milieu of added supportive therapeutic components which promote for instance cellular repair and normalization of function; their generally non‐immunogenic properties as the molecular contents are protected in physiological settings within lipid bilayers, eluding immune recognition; elevated uptake of their secretory components, as carried in extracellular vesicles owing to their recognition as biological carriers, optimizing delivery efficiency compared with non‐cellular/non‐vesicular molecular therapies; and the sustained secretion of therapeutically relevant factors following administration, which allows for a more protracted delivery within the recipient following each dose while simultaneously removing the need for higher and less well‐tolerated single‐dose concentrations within a single dose to achieve efficacy.

**TABLE 1:** Candidates for cell‐based therapies

Cell type	Candidates	Advantages	Disadvantages
Progenitor cells	Endothelial Progenitor Cells	High therapeutic efficacy	Difficult to isolate
	Epithelial Progenitor Cells	High therapeutic efficacy	Difficult to isolate
Stem cells	Embryonic Stem Cells	Totipotent	Tumorigenicity, ethical issues
	Induced Pluripotent Stem Cell	Accessibility, low rejection	Tumorigenicity, low efficacy
	Mesenchymal Stromal/Stem Cells	Accessibility, high therapeutic efficacy	Questionable immunogenicity, Controversial tumorigenicity
	Neural Stem/Progenitor Cells	Administration convenience, high compatibility with various treatment, low immunogenicity	Related research has not been found

Mesenchymal stem cells (MSCs) have been given particular attention in recent years as they do not require de‐differentiation as is the case with pluripotent sources, yet can still be induced to lineage‐specific, directed differentiation.^40^, ^41^ MSCs can bypass the technical constraints presented by isolating cells from specific organs, or ethical concerns surrounding use of embryonic cells because, they can be harvested from both autologous and allogeneic sources of relatively accessible tissue sources including umbilical cord, bone marrow, adipose tissue, and placenta.^42^, ^43^, ^44^ Careful characterization of these cells, including matching major histocompatibility complex (MHC) and genetic stability testing, may allow the development of a clinical‐grade, ready‐for‐use, allogeneic cell bank sourced from healthy donor stem cells to facilitate prompt administration of MSCs for acute diseases, circumventing time‐sensitive restrictions and technical constraints on the extraction and processing of patient‐derived tissues in point‐of‐care settings that would be required for autologous administration.^45^

Another potential stem cell candidate for ARDS‐targeted therapeutics is neural stem/progenitor cells (NSCs). NSCs have been widely investigated in studies of neurodegenerative diseases and cancers, but at present, studies utilizing NSCs in the context of ARDS are lacking.^46^, ^47^ Human foetal telencephalon‐derived NSCs have shown a positive clinical safety profile including low immunogenicity, low risk of tumourigenicity attributed to limited post‐transplantation proliferative activity, demonstrated the absence of lung aggregation or embolus formation, and relative technical ease of isolation and expansion compared with other types of stem cells.^48^ Indeed, these cell lines have already been applied by the authors in a number of completed or ongoing approved clinical trials in the United States, including NCT01172964, NCT02015819, and NCT03072134 (completed); and NCT02192359, NCT05139056 (ongoing/recruiting). NSCs have also displayed high compatibility with a variety of therapeutic agents, such as pro‐drug converting enzymes, oncolytic viruses, antibodies, oligonucleotides and nanoparticles,^47^, ^49^, ^50^, ^51^, ^52^, ^53^, ^54^, ^55^, ^56^, ^57^, ^58^, ^59^, ^60^ and they demonstrate inherent tropism migrating to sites of inflammation post‐injection. Using an avian v‐Myc transformation strategy, NSCs have also successfully been immortalized allowing for continued proliferation and expansion in culture, while maintaining a non‐tumourigenic and clinically safe profile as the v‐Myc‐transformed cells cease proliferation upon introduction to a host physiological environment, and are completely degraded by the host within several days post‐administration. This unique strategy provides opportunities for the formation of a therapeutically viable neural stem cell bank for wide and rapid allogeneic use.

More rigorous and comprehensive comparative studies of MSCs and NSCs evaluating their therapeutic contents, biodistribution, cytokine profiles, safety profiles and efficacy for central and peripheral pathological states will be needed to further investigate their potential as therapeutic interventions.^43^, ^44^, ^55^, ^56^ A summary of key examples from the basic science/preclinical literature investigating efficacy of MSC therapies in in vivo models of ARDS is presented in Table 2. Summarily, these collective studies provide a compelling body of preclinical evidence supporting the efficacy of therapeutic benefit in the attenuation and/or reversal of ARDS‐induced inflammation and lung histological damage in response to stem cell‐based therapeutic intervention. An important exception is noted in the case of severe influenza‐associated ARDS, for which reported outcomes are variable (see i.e. Darwish et al. 2013, Gotts et al. 2014). It is noted that this may be attributed to the inherent limitations of murine severe influenza models, which involve a short duration of pathology and generally do not permit assessment of long‐term histological and functional recovery. It is also noted in these studies however that MSC‐secreted factors TSG‐6 and PGE_2_ may be deleterious in severe influenza specifically owing to its effect in the upregulation of COX‐2, a positive correlate of morbidity and mortality in severe influenza.

**TABLE 2:** Representative preclinical MSC studies for ARDS

Injury type	Reference	Experimental model	MSC source	Dose; route of administration; timing of treatment	Outcomes
LPS, E coli, Bleomycin, P.aeruginosa	Cardenes et al. (2019)	Adult Dorsett Cross sheep weighing 30–40 kg, IV 5 µg/kg LPS from E. coli 055:B5 (Sigma, St. Louis, Missouri, USA)	hMAPCs	10 × 10^6^ cells/kg IV or 1 × 10^6^ cells/kg EB; 1 h after LPS infusion	Improvement in arterial oxygenation. Broad systemic distribution via IV route, localized distribution via EB route
	Chien et al. (2012)	Male BALB/C mice, intratracheal 25 μg LPS	Orbital fat‐derived stem/stromal cells	3 × 10^5^ cells; IV; 20 min after injury	Significant reduction in pulmonary inflammation, decrease in total protein concentration and neutrophil counts in alveolar fluid, reduced endothelial and alveolar epithelial permeability, reduced neutrophil and macrophage infiltration
	Danchuk et al. (2011)	8–10‐week‐old female BALB/C mice, oropharyngeal 1 mg/kg LPS from E. coli 0111:B4	Adult hMSCs from the Center for the Preparation and Distribution of Adult Stem Cells	2.5 × 10^5^ cells; oropharyngeal aspiration; 4 h after LPS infusion and 30 min after first dose	Significantly reduced lung inflammation, expression of pro‐inflammatory cytokines, neutrophil counts and total protein in bronchoalveolar region. Reduction in pulmonary edema
	Gupta et al. (2007)	6–8‐week‐old male C57BL/6 mice, intratracheal 5 mg/ml LPS from E. coli 055:B5 b(Sigma‐Aldrich)	mMSCs from GFP+‐C57BL/6 mice	7.5 × 10^5^ cells; intratracheal; 4 h after LPS infusion	Increased survival. Significant decrease in pulmonary edema and bronchoalveolar protein/endothelial and alveolar epithelial permeability
	Horie et al. (2020)	adult male Sprague Dawley rats, intratracheal 2 × 10^9E^. coli E5162 (serotype: O9 K30 H10) in a 300‐μL PBS suspension	Bone marrow and umbilical cord‐derived hMSC and UC‐derived CD362+ hMSC	1 × 10^7 cells/kg; IV; 30 min after E. coli instillation	Improved oxygenation. Reduced acute lung/histological injury, bacterial load, and inflammatory marker levels
	Jung et al. (2019)	7‐week‐old male C57BL/6 mice weighing 21–23 g, intratracheal 5 mg/kg LPS from E. coli 055:B5 (Sigma‐Aldrich, MO, USA)	Human adipose‐derived stem cells (hASCs) (StemPRO^®^ Human Adipose‐Derived Stem Cells; Thermo Fisher, MA, USA);	2 × 10^5^ cells; IV; 4 h after LPS infusion	Reduced neutrophil infiltration and myeloperoxidase levels. Reduced alveolar hemorrhage/congestion, lung injury scores, and collagen deposition around the vessels. Reduced levels of fibrosis accompanied by alveolar septal or interstitial thickening
	Li et al. (2019)	6–8‐week‐old male C57BL/6 mice weighing 20‐25g, intratracheal 50ul 2mg/ml LPS from E. coli 0111:B4 and 30 μl PBS 4 h after LPS infusion	mMSCs from Cyagen Biosciences, Inc. (Santa Clara, CA, USA), mMSCs‐short hairpin RNA (sh)control, mMSCs‐shLats1	5 × 10^4^ cells; airway; 4 h after LPS infusion	Reduced lung wet weight/body weight ratio, total bronchoalveolar fluid protein and albumin concentrations, and evidence of pulmonary fibrosis and pathological changes in lungs. Reductions in levels of proinflammatory factors, and increased levels of anti‐inflammatory factors. MSC differentiation toward alveolar type‐II epithelial cells observed
	Mao et al. (2015)	8–10‐week‐old male C57BL/6 mice, intratracheal 2 × 10^6^ CFU P. aeruginosa	mASCs	1 ×10^5^ cells or 1 × 10^6^ cells; intratracheal; 1 h after injury	Reduced bacterial burden, alveolar neutrophil accumulation, and reduced levels of myeloperoxidase, macrophage inflammatory protein−2 and total proteins in broncho‐alveolar fluid. Reduced evidence of lung injury
	Martinez‐Gonzalez et al. (2012)	10–12‐week‐old male BALB/C mice, intranasal 8 mg/kg LPS from E. coli 055:B5 (Sigma‐Aldrich)	hASCS or hASCs‐sST2	1 × 10^6^ cells; IV; 6 h after injury	Reduced lung airspace inflammation and vascular leakage, and evidence of preserved alveolar architecture. Significant reductions in protein content, differential neutrophil count, and proinflammatory cytokine concentrations in bronchoalveolar fluid. Absence of apoptosis and minimal inflammatory cell infiltration
	Rojas et al. (2005)	6–8 week old C57BL/6 mice; intratracheal 4 U/kg bleomycin	Bone marrow‐derived mMSCs	5 × 10^5^ cells; IV; 6 h after bleomycin administration	Differentiation of stem cells into specific and distinct lung cell phenotypes, increase in circulating levels of G‐CSF and GM‐CSF (known for their ability to promote the mobilization of endogenous stem cells), decrease in inflammatory cytokines
	Rojas et al. (2014)	Adult Dorsett Cross sheep weighing 36.5 to 65 kg, IV 3.5 μg/kg E. coli endotoxin LPS from E. coli 055:B5 (Sigma, St. Louis, MO, USA)	MultiStem (human bone marrow derived multipotent adult progenitor cells (hMAPCs))	4, 10, or 40 × 10^6^ cells; EB; 30 min after LPS infusion	Restoration of blood oxygen levels, improvement in carbon dioxide (CO2) clearance and pulmonary vascular pressure. Reduction in lung edema, reduced markers of inflammation
	Zhang et al. (2013)	8–10‐week‐old female C57BL/6 mice, oropharyngeal 15 mg/kg LPS from E. coli 055:B5 (Sigma‐Aldrich)	hASCs or mASCs isolated from inbred transgenic C57Bl/6‐Tg(UBC‐GFP)30Scha/J mice (Jackson Laboratories, Bar Harbor, ME, USA)	3.5 × 10^5^ cells; oropharyngeal aspiration; 4 h after injury and 30 min after first dose	Reductions in total protein and albumin concentrations in bronchioalveolar fluid and myeloperoxidase activity. Reduced leukocyte including neutrophil migration into alveoli, reduced expression of proinflammatory cytokines/increased anti‐inflammatory cytokine (IL−10)
Virus	Chan et al. (2016)	6–8‐week‐old female Balb/C mice, intranasal 106 TCID50 of H5N1 Influenza A/HongKong/486/97	Bone marrow‐derived hMSCs from the Texas A&M Health Science Center	5 x 10^5 cells; IV; 5 days post‐infection	Reversed infection‐induced downregulation of sodium and chloride transporter proteins associated with alveolar fluid clearance disruption. Reduced wet‐to‐dry lung weight ratio, vascular protein leakage/alveolar protein permeability. Reductions in inflammatory cytokine/chemokine levels and invading macrophages/monocytes in lung
	Darwish et al. (2013)	7–10‐week‐old male C57BL/6 mice, intranasal 425 EID50 or 150 EID50 influenza A/Mexico/4108/2009 (mouse‐adapted H1N1) or 1000 EID50 influenza A/Mexico/4108/2009 (swine‐origin pandemic H1N1)	bone marrow‐derived murine MSCs (mMSCs) and allogeneic hMSCs	2 × 10^5^ cells; IV; 4 h prior to infection and 2 days post‐infection or 2 and 5 days post‐infection	Negative outcome: Failure to improve survival or decrease pulmonary inflammation/inflammatory cell counts in influenza virus‐infected mice with or without combination with oseltamivir
	Gotts et al. (2014)	8‐week‐old female C57BL/6, intranasal 100 foci‐forming units of influenza A/H1N1/PR8	mMSCs and hMSCs from the National Institutes of Health repository in Temple, TX	5 × 10^5^ cells; retro‐orbital; 5 and 6 days after infection	Negative outcome: Failure to improve weight loss, lung water measures, markers bronchoalveolar inflammation, or histological pathological markers. However, prevention of influenza‐induced thrombocytosis modest reduction in lung viral load were observed
	Li et al. (2016)	6–8‐week‐old C57BL/6, intranasal 1 × 104 MID50 of A/HONG KONG/2108/2003 [H9N2 (HK)] H9N2 virus	bone marrow‐derived murine MSCs (mMSCs)	1 × 10^5^ cells; IV; 30 min pot‐infection	Significantly reduced proinflammatory chemokine and cytokine levels in lung. Reduced invading/inflammatory immune cell invasion in lungs. Improvements in lung histopathology and arterial blood gas observed
	Loy et al. (2019)	6–8‐week‐old female BALB/C mice, intranasal 106 log TCID50 of A/Hong Kong/486/1997(H5N1)	UC‐MSCs	5 × 10^5^ cells; IV; 5 days post‐infection	Restored alveolar fluid clearance, protein permeability measures. Modest improvement in survival

### 3.2 Therapeutic benefits of MSCs

The initiating events for the progression of acute lung injury and ARDS, whether caused by trauma, infection, or other mechanisms of immune dysregulation, involve the infiltration of neutrophils and macrophages to the alveolar space.^9^ These produce pro‐inflammatory cytokines, including TNF‐α and IL‐6, IL‐1β, and IL‐8, factors which amplify the production of ROS and severely damage endothelial and epithelial tissues by decreasing lung barrier function while increasing vascular permeability.^57^

**FIGURE 1:**
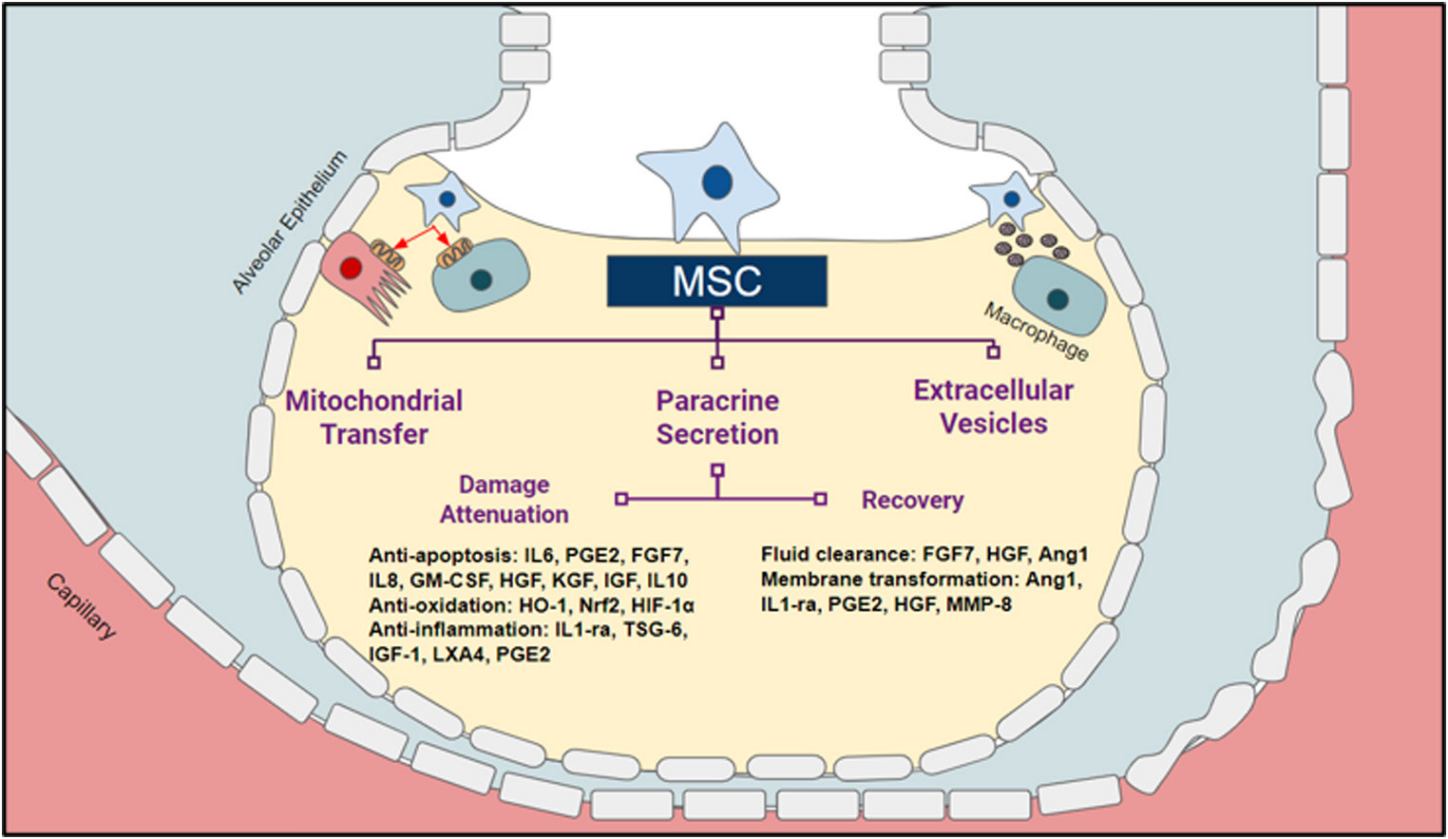
Mechanism of action for MSC therapy against ARDS. MSCs restore damaged lung tissue by secreting paracrine factors, transferring mitochondrial DNA, and liberating microvesicles. Secreted paracrine factors restore the alveolar cells by —first— reducing the effect of apoptosis, oxidation, and inflammation, and —second— restoring the fluid buildup and damaged tissues

MSCs act via secretion of multiple paracrine factors, either transported in extracellular vesicles or in a free state, including microRNAs, mRNAs, peptides^58^ and even mitochondrial DNA.^59^ Currently identified MSC secretory factors include the following: keratinocyte growth factor (KGF), interleukin‐1 receptor antagonist (IL1‐ra), TNF‐α‐stimulated tumour necrosis factor inducible gene 6 (TSG‐6), insulin‐like growth factor‐1 (IGF‐1), lipoxin A4 (LXA4), angiopoietin‐1 (Ang1), prostaglandin E_2_ (PGE‐2) and fibroblast growth factor 7 (FGF‐7).^54^, ^60^, ^61^, ^62^, ^63^, ^64^, ^65^, ^66^, ^67^, ^68^, ^69^, ^70^, ^71^ These paracrine factors, following uptake by recipient host cells, facilitate a variety of functions including alveolar fluid clearance, restore cell permeability, enhance resident immune cell phagocytosis, and enhance tissue repair in the lungs through anti‐inflammatory and anti‐apoptosis effect (Figure 1).^59^

#### 3.2.1 Anti‐apoptotic, anti‐oxidative, and anti‐inflammatory effects

MSCs demonstrate inflammation‐ and oxidative‐suppressive functions.^58^, ^59^, ^60^, ^61^, ^69^, ^70^ Hypoxic preconditioning has been shown to enhance this effect when transplanted to hypoxic sites, in addition to improving the survival, retention, proliferation and tissue formation of the damaged tissue.^72^, ^73^, ^74^ MSCs that overexpress cytoprotective factors such as heme oxygenase‐1 (HO‐1), nuclear factor erythroid 2‐related factor 2 (Nrf2), and hypoxia‐inducible factor 1‐alpha (HIF‐1α) also reduce oxidative stress‐induced cytotoxicity and apoptosis.^75^, ^76^, ^77^, ^78^ In addition to reducing oxidative stress, MSCs retain the chemotaxis and phagocytic activity of neutrophils, which facilitate pathogen clearance with reduced cytotoxicity to other immune cells and pulmonary cells in sites of inflammation sites.^69^ Patients with pathogen‐induced ARDS may especially benefit from this activity. In short, MSCs integrate neutrophil inhibition and reduction of oxidative stress by modulating secretion of both anti‐apoptotic and anti‐oxidative factors.

Suppression of the inflammatory response is by definition a form of immune‐suppression and would thus typically represent a contraindication for patients susceptible to or who have recently acquired a viral infection. Paradoxically, however, in COVID‐19 patients who have progressed to pneumonia and/or acute respiratory distress syndrome (ARDS), there is evidence that suppression of at least key aspects of an excessive immune/inflammatory response is conducive to improved survival. Indeed, some success has been found in COVID‐19 associated ARDS using the steroidal drug methylprednisolone, which also attenuates the inflammatory response.^79^

Mesenchymal stem cells have also paradoxically been shown to secrete a number of cytokines with anti‐apoptotic properties (e.g. IL‐6, IL‐8, GM‐CSF).^80^, ^81^ The oxidative stress results from persistent ROS production and ROS‐dependent NETosis (or neutrophil extracellular trap, where extracellular fibres are released to bind the pathogens, during the time of cell death) of activated neutrophils that inhibited from apoptosis because of the presence of anti‐apoptotic cytokines.^59^, ^70^, ^71^ These aspects of the MSC secretome might be hypothesized to promote ARDS pathogenesis by contributing to increased oxidative stress, and thus to act counter‐productively as an ARDS therapeutic. However, the demonstrated preclinical efficacy of MSCs in ARDS models suggests that the net effect of MSCs is overall suppression of inflammatory‐ARDS; further, it has been noted that these anti‐apoptotic cytokines may exhibit other therapeutically beneficial activities independent of inflammatory/oxidative modulation, such as prolonging the metabolic life of beneficial immune cells.^60^

The inflammation‐suppressing effects of MSCs are well demonstrated in in vivo literature. Adipose MSC‐derived paracrine factors have been demonstrated to inhibit T‐cell differentiation and activation as well as suppressing production of IFN‐y in vitro.^69^ Their secreted vesicles are already being tested in five currently registered clinical trials for respiratory distress via aerosolized inhalation (NCT04602104, NCT04602442, NCT04313647, NCT04276987, NCT04491240). Patient case studies/trials in Wuhan, China suggest that intravenously injected mesenchymal stem cells (MSCs) specifically may exhibit efficacy in attenuating symptoms once patients diagnosed with COVID‐19 have progressed to pneumonia. In a study by Leng and colleagues,^82^ this therapeutic approach was found to produce marked symptom improvements in seven hospitalized COVID‐19/pneumonia patients within days. In a related case study, a similar treatment approach was found to generate comparable improvements within two weeks in a 65‐year‐old patient after other mainstay therapies had failed.^83^ MSC therapies are already being tested in COVID‐19 patients on this pretext in a number of currently registered clinical trials (NCT04493242, NCT04384445, NCT04376987, NCT04371393, NCT04611256, NCT04903327, NCT04905836, NCT04615429, NCT04525378, NCT04355728, NCT04269525, ChiCTR2000031494, NCT04355728, ChiCTR2000029990, NCT04252118, NCT04269525, NCT04273646, NCT04288102, NCT04313322, NCT04302519, NCT04315987).

Hypoxic preconditioning has also been shown to enhance the anti‐oxidative capacity of MSCs when they are transplanted to hypoxic sites, in addition to improving the survival, retention, proliferation and tissue formation of the damaged tissue.^72^, ^73^, ^74^ MSCs that overexpress cytoprotective factors such as heme oxygenase‐1 (HO‐1), nuclear factor erythroid 2‐related factor 2 (Nrf2) and hypoxia‐inducible factor 1‐alpha (HIF‐1α) are effective in reducing oxidative stress‐induced cytotoxicity and apoptosis.^75^, ^76^, ^77^, ^78^ Moreover, MSCs have been shown to promote the chemotactic and phagocytic activity of neutrophils, which selectively facilitate pathogen clearance while avoiding cytotoxicity to host immune and pulmonary cells in sites of inflammation.^69^ Thus, MSCs integrate neutrophil inhibition and reduction of oxidative stress by modulating secretion of both anti‐apoptotic and anti‐oxidative factors.

The therapeutic efficacy of mesenchymal cells such as those of placental origin is not limited to suppression of inflammation. For example, secretory signals from trophoblast‐derived cells, in particular therapeutically relevant microRNAs, have been demonstrated to exhibit potent antiviral properties in in vitro assays.^84^

#### 3.2.2 Alveolar fluid clearance and restoration of cell permeability

Infiltration of neutrophils and pro‐inflammatory immune responses cause endothelial and epithelial cells to become permeable, resulting in pulmonary oedema.^57^ In the presence of damaged pulmonary tissues, MSCs secrete the keratinocyte growth factor FGF‐7, which contributes to restoring amiloride‐dependent sodium transport and enhancing alveolar fluid clearance.^67^, ^68^ Also contributing to clearing alveolar fluid, MSCs secrete cell ligands KGF and Ang‐1 that repair the membrane channels and their permeability.^65^, ^66^ Ang‐1 also acts as a stabilizing, anti‐inflammatory, and anti‐permeable agent to restore the plasma leakage and membrane insulation during the time of lung injury.^85^, ^86^, ^87^, ^88^ An in vitro study of introducing damaged alveolar epithelial cells (AECs) in different culturing environment showed that MSCs produce drastic increase of the IL‐1 receptor antagonists and prostaglandin E_2_ in the presence of the injured AECs and these factors re‐established the normal permeability of the epithelial cells.^54^ This experiment demonstrates the possible route of action of MSCs when administered into patients with injured lung. Thus, administration of MSCs has potential to re‐establish normal alveolar fluid level in patients with pulmonary oedema by removing fluid and restoring cell permeability.

#### 3.2.3 Anti‐inflammatory responses

High levels of pro‐inflammatory cytokines, such as TNFα, TGFβ1, IL‐1β, and IL‐8, have been reported in pulmonary oedema fluid from ARDS patients.^89^ TNFα and IL‐1β are among a cocktail of cytokines released from macrophages after the immune system is activated.^90^ Once released, they act as specific cell‐membrane bound receptors to activate a signalling cascade to increase production of pro‐inflammatory cytokines, lipid mediators, ROS and cell adhesion molecules.^90^, ^91^ The increased production of cytokines, lipid mediators and cell adhesion molecules facilitates migration of the inflammatory cells into tissues and worsens the lung injury as a result.^90^ In an in vitro study, the MSCs secreted cytokine IL‐1RA, which dramatically lessens the inflammation effect of TNFα and IL‐1β expressed by macrophages.^54^ IL‐1RA acts as a competitive inhibitor to IL‐1β by blocking IL‐1β’s binding site and the production of TNFα by macrophages.^54^ MSCs also release substances such as TSG‐6, IGF‐1 and LXA4, which induce anti‐inflammatory responses in murine acute lung injury models by directly acting on the cells inducing inflammation to undergo phenotypic transition.^57^, ^61^, ^62^, ^63^, ^64^, ^92^, ^93^ In addition to abrogating the effect of TNF‐α, IL‐6, and IL‐1β, MSCs have also been shown to inhibit recruitment of neutrophils and protein formulation within the inner alveolar space.^94^

MSCs can also act to reduce cell attraction and migration as mediators of inflammatory processes, as in lung injury settings. For example, TGFβ1 has an essential role in lung repair and fibroproliferation by promoting collagen synthesis.^95^ However, overexpression of TGFβ1 has been shown to facilitate the migration of fibroblasts from the extracellular space into the intracellular alveolar space, and to activate the human procollagen I promoter to induce inflammation of lung tissues.^96^, ^97^ IL‐8 is also involved in regulating the migration of endothelial cells into the alveolar space.^98^ Moreover, the anti‐IL‐8 autoantibody, which binds to IL‐8 with a very high affinity and prevents IL‐8 from attracting and binding to neutrophils, forms a complex with IL‐8 and immunoglobulin G, recruiting an inflammatory response from the periphery.^99^, ^100^, ^101^, ^102^

#### 3.2.4 Genetic modification of stem cell therapies

Because inflammatory pathway induction is the key underlying cause of ARDS, extensive studies involving genetic modifications of MSCs have been conducted to improve the anti‐inflammatory properties of MSCs. One system that has been a focus for genetic modification approaches in the context of cardiovascular diseases and is receiving increasing attention in the study of ARDS, is the renin‐angiotensin system (RAS). Within the RAS, there is substantial interest arises in the angiotensin‐converting enzyme (ACE). The ACEs cleave the peptide hormone angiotensin‐I (Ang I) into Ang II. Ang II interacts with angiotensin II type 1 receptor (AT1R) and induces pulmonary vasoconstriction and increases vascular permeability, leading to oedema. Ang II also exerts pro‐inflammatory effects and promotes fibroproliferation.^103^, ^104^ On the other hand, ACE2 degrades Ang II to Ang‐(1–7), which interacts with the Mas receptor to mediate anti‐inflammatory responses. Because the type II alveolar epithelial cells, where ACE2 is produced, are severely damaged in ARDS,^103^ the ACE2/Ang‐(1–7)/Mas axis is an interesting target for genetically modifying MSCs to overexpress ACE2 to counter the aggravated effects of the ACE/AngII/AT1R axis in ARDS. MSCs that overexpress ACE2 have shown therapeutic benefits in reducing neutrophil influx and pro‐inflammatory cytokine production in preclinical studies, and hence promoting endothelial repair and rescuing pulmonary functions.^105^, ^106^

Relevant to COVID‐19 and ARDS, transmembrane ACE2, together with the cellular serine protease TMPRSS2, mediates cell entry of SARS‐CoV‐2 and its in vivo replication.^107^, ^108^, ^109^ This raises the question of whether ACE2 expression by administered stem cells could complicate treatment of SARS‐CoV‐2 by allowing for infection of administered cells. A clinical trial conducted in China showed that transplanted MSCs express minimal gene expression of ACE2 and TMPRSS2, the two main routes of infection for SARS‐Cov‐2.^82^, ^108^, ^109^, ^110^ These MSCs also secrete high levels of immunomodulatory factors (e.g. IL‐10, IP‐10, TNF‐α, TGF‐β, HGF, LIF, GAL, NOA1, FGF, VEGF, EGF, BDNF, NGF) after transplantation and stimulate lung repair, improving clinical outcomes for patients.^82^ These contradictory findings regarding cytokine production in ACE2‐overexpressing verses ACE2‐negative MSCs indicate the urgent need to identify targets other than the ACE2 axis to maximize the benefits of genetically modified MSCs in treating ARDS, whether induced by SARS‐CoV‐2 or not. In addition to ACE2 overexpression or knockout MSCS, MSCs coadministered with human recombinant soluble ACE2 (hrsACE2) have been evaluated. A recent cryoelectron microscopic study of SARS‐CoV‐2 claimed that these MSCs inhibit SARS‐CoV‐2 infections in host cells, and they can prevent downstream effects of COVID‐19 during an early stage.^111^ Although the therapeutic benefits of hrsACE2‐cotreated or overexpressing MSCs are not known during later stages of COVID‐19, hrsACE2‐expressing MSCs may still show effects similar to those of ACE2‐negative MSCs in terms of blocking SARS‐CoV‐2 cell entry and replication and stimulating lung repair through undiscovered pathways.

Substantial emphasis has also been put on another inflammatory signalling pathway, the IL‐33/ST2 pathway. IL‐33, a recently discovered member of the IL‐1 cytokine family, is abundantly present in endothelial and epithelial cells in the skin, gastrointestinal tract and lungs. It is released as an alarmin during injury and interacts with its receptor, suppression of tumourigenicity 2 (ST2).^112^, ^113^, ^114^, ^115^, ^116^ ST2 is expressed in two isoforms, a transmembrane receptor (ST2L) and a soluble decoy receptor (sST2). Interaction of IL‐33 with these isoforms triggers opposing inflammatory signalling pathways.^114^, ^117^, ^118^ IL‐33/ST2L initiates acute inflammation through Th2‐dependent immune responses, while IL‐33/sST2 attenuates Th2‐dependent inflammation as sST2 lacks the intracellular Toll/Interleukin‐1 receptor domain to induce the signalling pathway.^117^, ^119^ The sSTR2‐overexpressing MSCs showed therapeutic benefit in mice in attenuating acute LPS‐induced pulmonary inflammation.^120^ In addition, the plasma concentration of sST2 can be used as a diagnostic factor to distinguish ARDS from acute heart failure and serves as a prognostic biomarker to assess the severity of ARDS to determine how supportive treatments and weaning practices should be implemented.^121^, ^122^, ^123^

In addition to their anti‐inflammatory properties, the therapeutic efficacy of MSCs depends on several other factors such as homing, tissue restoration and protective effects against apoptosis and oxidation. Genetic modification can thus serve as an excellent tool to improve the therapeutic benefits of MSCs. MSCs have shown tropism to injury sites, but they may lose homing receptors after the large‐scale expansion needed to produce enough cells for therapeutic doses.^124^ MSCs genetically engineered to overexpress the C‐X‐C motif chemokine receptor 4 (CXCR4) on their cell surface showed improved homing to sites of tissue injury. CXCR4 interacts with its ligand, stromal cell‐derived factor‐1 (SDF‐1), which has elevated expression at sites of tissue injury.^125^, ^126^, ^127^, ^128^ Similarly, MSCs that overexpress the E‐prostanoid 2 (EP2) receptor, which interacts with prostaglandin E2, have shown improved homing and retention.^129^, ^130^ EP2‐overexpressin MSCs have also shown additional benefits in terms of tissue restoration by reducing pulmonary vascular permeability and improving histopathology.^130^ Alveolar restoration and oedema clearance have been improved by use of MSCs that overexpress KGF. KGF plays a significant role in stimulating proliferation of alveolar type II cells and surfactant synthesis for pulmonary epithelial repair.^67^, ^131^ To further enhance lung repair and restore pulmonary functions, genetic modifications involving anti‐apoptosis and anti‐oxidation pathways, for example overexpression of heme oxygenase‐1, are also being extensively investigated.^75^, ^116^, ^132^

These studies of diverse genetic modifications to MSCs have shown that a single modification can yield multiple benefits and suggest promise of this approach. Given the rapid advance of genetic engineering technology to date, we may expect that the huge capacity of MSCs will allow multiple modifications, raising the possibility of synergistic benefits to treat ARDS and increased numbers of genetically modified MSCs translated to clinical studies.

## 4 CURRENT CLINICAL TRIALS USING MSCS TO TREAT ARDS

Due to the relative ease of tissue/cell sourcing and the consequently higher volume of preclinical work investigating clinical viability, MSC therapies have been the subject of a relatively higher number of approved clinical trials compared with other stem cell‐based therapeutics. As of 5 January 2022, there are 65 and 122 interventional stem cell clinical trials for ARDS and COVID‐19, respectively, listed on clinicaltrials.gov. Han and colleagues note the key limitation that many of the present studies in this area are limited by small sample sizes and lack of a well‐defined time‐response relationship between MSC administration and patient performances.^125^

Current registered trials using stem cell interventions for COVID‐associated and other presentations of ARDS are summarized in Tables 3 and 4.

**TABLE 3:** Examples of Interventional Stem Cell Clinical Trials for ARDS as of October 7, 2021

Identifier	Status	Phase	Treatment	Dose		Regimen (total number of doses; frequency)	Route	Country
				Cells	Cells/kg			
NCT05127122	Not yet recruiting	I/II	ExoFlo	10 ml or 15 ml		Single dose		USA
NCT04347967	Not yet recruiting	I	UMC119‐06	Low, medium, and high		Single dose	IV	Taiwan
NCT04371393	Recruiting	III	Remestemcel‐L	‐	2 × 10^6^	Two doses; four days apart	IV	USA
NCT04366063	Recruiting	II/III	MSCs or MSCs +MSC‐EVs	100 × 10^6^ (±10%) w/o EVs 100 × 10^6^ (±10%) w/ Evs	‐	Two doses; Day 0 and 2 (MSCs), Day 4 and 6 (MSC‐EVs)	IV	Islamic Republic of Iran
NCT04367077	Recruiting	II/III	MultiStem	N/A	N/A	IV	USA	
NCT02804945	Completed	II	Allogeneic Human MSCs	‐	maximum 3 × 10^6^	Single dose	IV	USA
NCT02112500	Unknown (previously recruiting)	II	MSCs cultured and extracted from bone marrow of enrolled patients	N/A	N/A	IV	Korea	
NCT04348461	Not yet recruiting	II	Allogeneic and expanded adipose tissue‐derived mesenchymal stromal cells	‐	1.5 × 10^6^	Two doses; N/A	IV	Spain
NCT03807804	Recruiting	II	HLCM051 (MultiStem)	900 × 10^6^ (±20%)	‐	Single dose	IV	Japan
NCT04377334	Not yet recruiting	II	Allogeneic BM‐MSCs	N/A	N/A	N/A	Germany	
NCT02444455	Unknown (previously recruiting)	I/II	Human Umbilical‐Cord‐Derived MSCs (UCMSC)	‐	0.5 × 10^6^	Three doses; once a day	IV	China
NCT04289194	Active, not recruiting	I/II	HCR040 (whose active substance is HC016, allogeneic adipose‐derived adult mesenchymal stem cells expanded and pulsed with H_2_O_2_)	‐	1 × 10^6^ 2 × 10^6^	Phase I: Single dose; dose escalation Phase II: Single dose of 2 × 10^6^ cells/kg	IV	Spain
NCT02095444	Unknown (previously recruiting)	I/II	Menstrual blood stem cells	‐	10 × 10^6^	Four doses; two doses per week and two weeks	IV	China
NCT04355728	Recruiting	I/II	Umbilical Cord Mesenchymal Stem cells (UCMSC)	100 × 10^6^	‐	Two doses; within 24 and 72 h	IV	USA
NCT03042143	Recruiting	I/II	Realist Orbcel‐C (Human umbilical cord derived CD362 enriched MSCs)	100 × 10^6^ 200 × 10^6^ 400 × 10^6^	‐	Phase I: Single dose; Dose escalation Phase II: Single dose of 400 × 10^6^ cells	IV	USA
NCT04331613	Recruiting	I/II	CAStem (immunity‐ and matrix‐regulatory cells (IMRCs), also named M cells, differentiated from clinical‐grade human embryonic stem cells (hESCs))	‐	3 × 10^6^ 5 × 10^6^ 10 × 10^6^	Single dose; dose escalation	IV	China
NCT04390139	Recruiting	I/II	XCEL‐UMC‐BETA (Wharton‐Jelly MSCs)	‐	1 × 10^6^	Two doses; Day 1, 3	IV	Spain
NCT02611609	Completed	I/II	MultiStem (adult stem cell)	Low High	‐	Single dose, dose escalation	N/A	USA
NCT04333368	Recruiting	I/II	Umbilical cord Wharton's jelly‐derived human	‐	1 × 10^6^	Three doses; Day 1, 3, 5	IV	France
NCT02175303	Unknown	I/II	Placenta‐derived decidual stromal cell therapy	‐	1 × 10^6^	One or more doses; Weekly	IV	Sweden
NCT01775774	Completed	I	Allogeneic Bone Marrow‐Derived Human MSCs	‐	1 × 10^6^ 5 × 10^6^ 10 × 10^6^	Single dose; dose escalation	IV	USA
NCT04390152	Not yet recruiting	I	Wharton's jelly derived Mesenchymal Stem cells	50 × 10^6^	‐	Two doses; N/A	IV	Colombia
NCT01902082	Unknown (previously recruiting)	I	Allogeneic Adipose‐derived MSCs	‐	1 × 10^6^	Single dose	IV	China
NCT04347967	Not yet recruiting	I	Human umbilical cord‐derived MSCs (UMC 119–06)	Low Medium High	‐	N/A	IV	Taiwan
NCT04400032	Not yet recruiting	I	BM‐MSCs	25 × 10^6^ 50 × 10^6^ 90 × 10^6^	‐	Three doses, consecutive days (dose escalation)	IV	Canada
NCT04345601	Not yet recruiting	I	BM‐MSCs	‐	2 × 10^6^	Single dose	IV	USA
NCT03608592	Recruiting	N/A	Human Umbilical Cord MSCs	‐	1 × 10^6^	Single dose	IV	China

Note

Search parameters: Condition or disease: Acute Respiratory Distress Syndrome. Other terms: Stem cell. Excluded studies: Observational studies, non‐stem cell interventions.

**TABLE 4:** Examples of Interventional Stem Cell Clinical Trials for COVID‐19 as of 05 January 2022

Identifier	Status	Phase	Treatment	Dose		Regimen (total number of doses; frequency)	Route	Country
				Cells	Cells/kg			
NCT05132972	Recruiting	II/III	UCMSCs		1 × 10^6^	Three doses; Day 0, Day 3, and Day 6	IV	Indonesia
NCT04490486	Not yet recruiting	I	UCMSCs	100 × 10^6^		Two doses; Day 0, 3	IV	USA
NCT04371393	Recruiting	III	MSCs (Remestemcel‐L)	‐	2 × 10^6^	Two doses; Four days apart	IV	USA
NCT04366063	Recruiting	II/III	MSCs or MSCs +MSC‐EVs	100 × 10^6^ (±10%) w/o EVs 100 × 10^6^ (±10%) w/ Evs	‐	Two doses; Day 0 and 2 (MSCs), Day 4 and 6 (MSC‐EVs)	IV	Islamic Republic of Iran
NCT04367077	Recruiting	II/III	MultiStem	N/A	N/A	IV	USA	
NCT04416139	Recruiting	II	Mesenchymal Stem cells	‐	1 × 10^6^	Single dose: Day 1	IV	Mexico
NCT04315987	Not yet recruiting	II	NestCell^®^ Mesenchymal Stem Cell	20 × 10^6^	‐	Three doses; Day 1, 3, 5	IV	Brazil
NCT04348435	Enrolling by invitation	II	Hope Biosciences Allogeneic Adipose‐derived Mesenchymal Stem Cell Therapy (allogeneic HB‐adMSCs)	50 × 10^6^ 100 × 10^6^ 200 × 10^6^	‐	Five doses; Week 0, 2, 6, 10, 14	IV	USA
NCT04349631	Enrolling by invitation	II	Autologous HB‐adMSCs	N/A	Five Doses; N/A	IV	USA	
NCT04288102	Recruiting	II	MSCs	40 × 10^6^	‐	Three doses; Day 0, 3, 6	IV	China
NCT04348461	Not yet recruiting	II	Allogeneic and expanded adipose tissue‐derived mesenchymal stromal cells	‐	1.5 × 10^6^	Two doses; N/A	IV	Spain
NCT04362189	Not yet recruiting	II	HB‐adMSCs	100 × 10^6^	‐	Four doses; Day 0, 3, 7, 10	IV	USA
NCT04299152	Not yet recruiting	II	Stem Cell Educator‐Treated Mononuclear Cells Apheresis (autologous human multipotent cord blood stem cells (CB‐SC)	N/A	Single (extra dose if needed); a week apart	IV	USA	
NCT04377334	Not yet recruiting	II	Allogeneic BM‐MSCs	N/A	N/A	IV	Germany	
NCT04389450	Not yet recruiting	II	PLX‐PAD (allogeneic ex vivo expanded placental mesenchymal‐like adherent stromal cells)	Interval high dose High dose Low dose	‐	Interval high dose: 15 doses; 1 week apart) High dose: Single dose Low dose: Single dose	Intramuscular	USA and Israel
NCT04361942	Recruiting	II	Allogeneic MSCs	‐	1 × 10^6^	Single dose	IV	Spain
NCT04269525	Recruiting	II	UC‐MSCs	99 × 10^6^	‐	Four doses; Day 1, 3, 5, 7	IV	China
NCT04336254	Recruiting	I/II	Allogeneic human dental pulp MSCs (BSD BTC & Utooth BTC)	30 × 10^6^	‐	Three doses; Day 1, 4, 7	IV	China
NCT04366323	Recruiting	I/II	Allogeneic and expanded adipose tissue‐derived MSCs	80 × 10^6^	‐	Two doses; N/A	IV	Spain
NCT04382547	Enrolling by invitation	I/II	Allogenic Pooled Olfactory Mucosa‐derived Mesenchymal Stem Cells	N/A	N/A	IV	Belarus	
NCT04346368	Not yet recruiting	I/II	Bone marrow‐derived MSCs	‐	1 × 10^6^	Single dose	IV	China
NCT04390152	Not yet recruiting	I/II	WJ‐MSCs	50 × 10^6^	‐	Two doses; N/A	IV	Colombia
NCT04339660	Recruiting	I/II	UC‐MSCs	‐	1 × 10^6^	Single dose	IV	China
NCT04392778	Recruiting	I/II	Allogeneic UC‐MSCs	‐	3 × 10^6^	Three doses; Day 0, 3, 6	IV	Turkey
NCT04355728	Recruiting	I/II	UC‐MSCs	100 × 10^6^	‐	Two doses; Day 1, 3	IV	USA
NCT04331613	Recruiting	I/II	CAStem	‐	3 × 10^6^ 5 × 10^6^ 10 × 10^6^	Single (Dose escalation)	IV	China
NCT04390139	Recruiting	I/II	XCEL‐UMC‐BETA (Wharton‐Jelly mesenchymal stromal cells)	‐	1 × 10^6^	Two doses; Day1, 3	IV	Spain
NCT04341610	Withdrawn	I/II	Allogeneic adipose tissue‐derived MSCs	100 × 10^6^	‐	N/A	N/A	Denmark
NCT04398303	Not yet recruiting	I/II	ACT−20‐MSC (allogeneic human umbilical derived mesenchymal stem cells) or ACT−20‐CM (ACT−20‐MSC conditioned medium)	‐	1 × 10^6^ cells/kg in 100 mL CM 100 mL CM only	N/A	IV	USA
NCT03042143	Recruiting	I/II	Realist Orbcel‐C (Human umbilical cord derived CD362 enriched MSCs)	400 × 10^6^	‐	Single dose	IV	UK
NCT04333368	Recruiting	I/II	umbilical cord Wharton's jelly‐derived mesenchymal stromal cells (UC‐MSC)	‐	1 × 10^6^	Three doses; Every other day	IV	France
NCT04313322	Recruiting	I	Wharton's Jelly Mesenchymal stem cells (WJ‐MSCs) derived from cord tissue of newborns	‐	1 × 10^6^	Three doses; three days apart	IV	Jordan
NCT04252118	Recruiting	I	MSCs	30 × 10^6^	‐	Three doses; Day 0, 3, 6	IV	China
NCT04302519	Not yet recruiting	I	Dental pulp mesenchymal stem cells	‐	1 × 10^6^	N/A; Day 1, 3, 7 (Dose escalation)	IV	China
NCT04371601	Active, not recruiting	I	UC‐MSCs	‐	10^6^	Four doses; every 4 days	IV	China
NCT04397796	Not yet recruiting	I	Allogeneic bone marrow‐derived MSCs (CD73+, CD90+, CD105+, CD14‐, CD34‐, CD45‐, HLA‐DR‐)	N/A	N/A	N/A	USA	
NCT04400032	Not yet recruiting	I	BM‐MSCs	25 × 10^6^ 50 × 10^6^ 90 × 10^6^	‐	Three doses; Three consecutive days (24±4 h apart) (dose escalation)	IV	Canada
NCT04345601	Not yet recruiting	I	Allogeneic blood‐derived MSCs	1 × 10^8^	‐	Single dose	IV	USA
NCT04273646	Not yet recruiting	N/A	Human Umbilical Cord MSCs	‐	0.5 × 10^6^	Four doses; Day 1, 3, 5, 7	IV	China
NCT04393415	Not yet recruiting	N/A	Cord blood stem cells or platelet rich plasma (PRP	N/A	N/A	N/A	Egypt	
NCT04293692	Withdrawn	N/A	UC‐MSCs	‐	0.5 × 10^6^	Four doses; Day 1, 3, 5, 7	IV	China

Note

Search parameters: Condition or disease: COVID; Other terms: Stem cell. Excluded studies: Observational studies, non‐stem cell interventions

## 5 CELL‐FREE THERAPIES DERIVED FROM THE STEM CELL SECRETOME: EXTRACELLULAR VESICLES AND MIRNA

In light of the key role of secretory factors in conferral of therapeutic benefits from MSC therapy, there is increasing interest in studying the therapeutic benefits of MSC‐derived extracellular vesicles (MSC‐EVs) as compared to MSCs themselves.^125^, ^126^, ^127^ As MSC‐EVs are a cell‐free treatment, they offer multiple advantages over cell‐based treatments.^133^ First, as they are non‐nucleated and acellular, they cannot proliferate, and thus, there is minimal risk of tumourigenicity.^134^ Second, as they do not express HLA antigens, they pose much lower risks of immunogenicity, and graft‐versus‐host‐disease than their source cells, and thus are safer for allogeneic transplantation as they pose a lower risk of host immunorejection.^135^, ^136^, ^137^ Third, MSC‐EVs are smaller than cells, which allows for better penetration into target tissues. In contrast, because of their larger dimensions cell therapies are restricted from penetrating certain membrane barriers or extravasating from capillaries to key tissues relative to their much smaller secretory vesicles,^137^, ^138^, ^139^, ^140^, ^141^ and they carry a higher risk of embolus formation.^142^, ^143^, ^144^, ^145^, ^146^, ^147^, ^148^ Furthermore, storage of MSC‐EVs does not require cryopreservatives, such as DMSO, which are necessary for long‐term storage of MSCs but are detrimental to their viability. In addition, MSC‐EVs are less affected by repeated freeze and thaw cycles as compared to MSCs.^136^, ^143^, ^149^, ^150^

**FIGURE 2:**
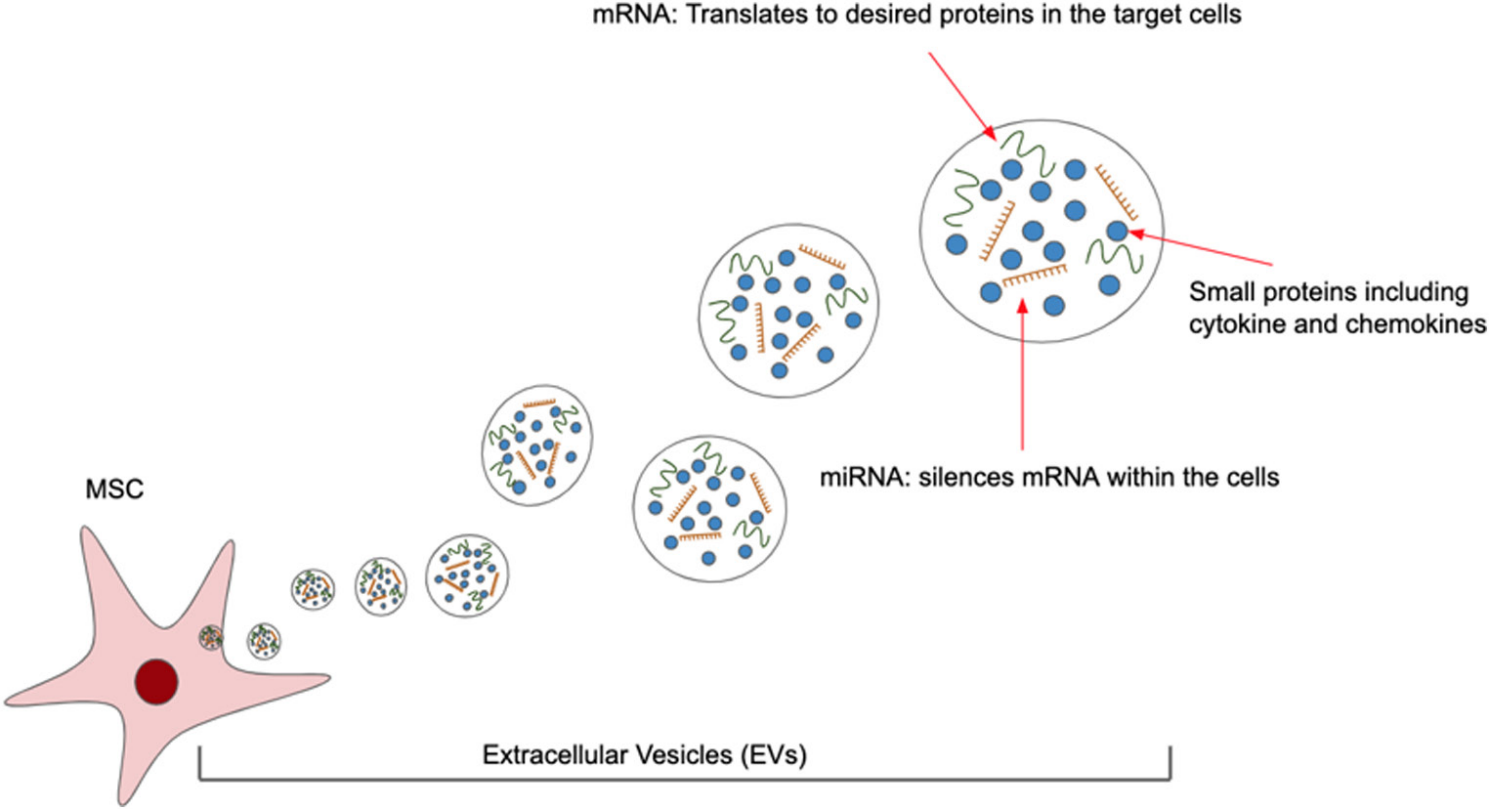
Secretome of MSC packaged by EVs. EVs produced by MSCs carry mRNA, miRNA, and small proteins that can act on target cells

The secretome of MSCs and other stem cells includes small soluble proteins such as cytokines, chemokines, growth factors, and anti‐inflammatory factors.^151^ MSC‐EVs contain microRNAs (miRNAs) and messenger RNAs (mRNAs) that interact with the target cell's endogenous mRNAs and facilitate the production of proteins with therapeutically relevant including inflammation‐suppressing niches within the cells (Figure 2).^136^, ^152^, ^153^, ^154^, ^155^ These various EV contents affect a variety of cell functions to bring desired effects in different disease models.^156^, ^157^, ^158^ EV‐based therapies have also been artificially engineered to incorporate key therapeutic components for delivery.^159^, ^160^

There is ample evidence that extracellular vesicles and their factors derived from a variety of cell sources are also able to directly impart immune‐modulatory effects on recipient cells and physiologic systems through their therapeutically relevant intrinsic contents. For example, dendritic cell (DC) exosomes reduced inflammation and associated arthritis in a murine model.^161^ Exosomes within the secretome of multiple immune cell types have themselves been found to participate in host immunity against invading viral pathogens by transporting antiviral factors between cells and activating antiviral mechanisms,^162^ directly inhibiting pathogen proliferation and infection as well as inducing humoral and cytotoxic immunity.^163^

It has been demonstrated for instance that murine bone marrow DCs pulsed with diphtheria toxoid (DT) are induced to generate exosomes promoting a DT‐specific immunoglobin response.^164^ Treatment with exosomes derived from *Toxoplasma gondii* antigen (Ag)‐pulsed DC cells was similarly shown to induce anti‐*T. gondii* Ag antibodies in association with elevated humoral response and symptom improvement in infected mice.^165^ Infection with *Mycobacterium tuberculosis* promoted release of exosomes containing *M. tuberculosis* MHC‐II complexes associated with antimicrobial activity from murine macrophages.^166^ Exosome anchoring protein‐fused DNA vectors expressing antigens specific to Human Papilloma Virus (HPV)^167^ as well as a broad spectrum of other viruses including Influenza, Hepatitis C, Crimean‐Congo Hemorrhagic Fever, and the flaviviruses Ebola and West Nile Virus^168^ were effective in promoting an antigen‐specific cytotoxic T lymphotyte (CTL) response in mice. Moreover, exosomes loaded with ovalbumin antigens from DC pulsing have been shown to not only augment T‐cell responsivity, IFN‐y and IgG production but to mediate a Th1 shift.^169^

There is also evidence of secreted exosomes exhibiting directly antiviral properties through their intrinsic contents. Exosomes secreted by macrophages in response to IFN‐a have been demonstrated to utilize Hepatitis A receptors to deliver antiviral substances to hepatocytes.^170^ Exosomes isolated from human trophoblasts have also been found to exhibit directly antiviral properties in vitro, associated with miRNA cargoes derived from the chromosome 19 cluster as well as a unique peptide and phospholipid repertoire.^84^ Intriguingly, Herpes Simplex 1 (HSV‐1) viral microRNAs miR‐H28 and miR‐H29 transmitted via exosomes have also been found to restrict viral cell‐cell transmission via IFN‐y upregulation, postulated by the authors to represent a mechanism of limiting viral spread to uninfected cells in favour of maximizing transmission to an alternate host.^171^ Exosomes containing the spike S protein derived from other variants of SARS‐associated coronavirus (SARS‐CoV) have also been found to successfully induce the generation of neutralizing antibodies in a murine model^172^

The investigation of the therapeutic benefits of MSC‐EVs in comparison to MSCs is a rapidly developing field. As of 5 January 2022, there are nine MSC‐EV clinical trials, summarized in Table 5.

**TABLE 5:** Interventional MSC‐EV Clinical Trials as of January 05, 2022

Identifier	Status	Phase	Treatment	Dose		Regimen (total number of doses; frequency)	Route	Country
				Cells	Cells/kg			
NCT04602442	Enrolling by invitation	II	MSC‐EVs	0.5–2 × 10^10^ EVs /3 mL		Twenty doses; twice a day	Inhalation	Russia
NCT04491240	Completed	I/II	MSC‐EVs	0.5–2 × 10^10^ EVs /3 mL		Twenty doses; twice a day	Inhalation	Russia
NCT04276987	Completed	I	MSC‐EVs	2.0 × 10^8^ EVs /3 mL		5 doses; once a day	Inhalation	China
NCT04493242	Completed	II	MSC‐EVs	1X @ 8 or 12 × 10^11^/ 100 ml		Single dose	IV	USA
NCT04798716	Not yet recruiting	I/II	MSC‐EVs	2 × 10^9^ or 4 × 10^9^ or 8 × 10^9^ EVs /mL		Three doses; once every other day	IV	USA
NCT04366063	Recruiting	II/III	MSCs or MSCs +MSC‐EVs	100 × 10^6^ (±10%) w/o EVs 100 × 10^6^ (±10%) w/ EVs	‐	Two doses; Day 0 and 2 (MSCs), Day 4 and 6 (MSC‐EVs)	IV	Islamic Republic of Iran
NCT04398303	Not yet recruiting	I/II	ACT−20‐MSC (allogeneic human umbilical derived mesenchymal stem cells) or ACT−20‐CM (ACT−20‐MSC conditioned medium)	‐	1 × 10^6^ in 100 ml CM 100 ml CM only	N/A	IV	USA
NCT04276987	Not yet recruiting	I	Allogenic adipose mesenchymal stem cells derived exosomes (MSCs‐Exo)	2 × 10^8^ nano vesicles	‐	Five doses; Day 1, 2, 3, 4, 5	Aerosol inhalation	China
NCT04313647	Recruiting	I	A Tolerance Clinical Study on Aerosol Inhalation of Mesenchymal Stem Cells Exosomes In Healthy Volunteers	1X @ 2.0 × 10e8 ‐ 8.0 × 10e8 exosomes / dose		Single dose	Aerosol inhalation	China

## 6 CONCLUSION

The persistent emergence of novel SARS‐Cov‐2 variants, and with these fears of novel strains capable of effective vaccine escape and/or heightened virulence, combined with continued struggles in meeting global demands of accessibility for vaccines to local populations, underscore the clear need that remains for effective therapies to address SARS‐Cov‐2 infection and severe COVID‐19 clinical manifestation. Stem cell‐based therapy has great potential for treating COVID‐19 associated ARDS, a condition presenting a high mortality risk for which there remains a substantial unmet need for effective treatment interventions. Stem cell‐based therapies have immunomodulatory effects and multiple preclinical and clinical studies have supported their proposed efficacy against ARDS.^124^, ^127^, ^159^, ^171^, ^173^ Among the many types of stem cells currently being evaluated for ARDS and/or COVID‐19, MSC‐based therapies are gaining popularity because of their ease of isolation and convenient culturing.^43^ MSC‐EVs are also of increasing interest as an acellular alternative modality able to exert many of the key therapeutically relevant benefits without presenting the risks of cell therapies such as immunogenicity or tumourigenesis. Further clinical studies incorporating larger patient sample sizes are needed to more definitively establish optimal dose schedules and dose‐response times, and to better evaluate comparative efficacy with cell therapies derived from specific optimized culture conditions to maximize therapeutic contents and functional benefit, and from varying cell/tissue sources both mesenchymal and potentially investigating non‐mesenchymal alternatives of equal or superior therapeutic value for this indication. Moreover, stem cell‐derived secretory factors, as contained in extracellular vesicles, should be given greater attention as an acellullar alternative therapeutic niche presenting much of the benefit demonstrated preclinically and clinically with their source cells, but circumventing the technical and safety concerns presented by administration of cell as opposed to cell‐secretome based therapies.

## CONFLICT OF INTEREST

CDC is affiliated with StemXO Inc., a private biotechnology company with an interest in stem cell therapeutic development. The other authors declare no conflicts of interests.

## AUTHOR CONTRIBUTIONS

**Gary Ngai:** Investigation (equal); Writing – original draft (equal); Writing – review & editing (equal). **Dae Hong Kim:** Formal analysis (equal); Writing – review & editing (equal). **Mohamed A hammad:** Formal analysis (equal); Writing – review & editing (equal). **Margarita Gutova:** Formal analysis (equal); Writing – review & editing (equal). **Karen Aboody:** Conceptualization (equal); Writing – review & editing (equal). **Christopher D Cox:** Conceptualization (equal); Formal analysis (equal); Writing – original draft (equal); Writing – review & editing (equal).

## Data Availability

Data sharing not applicable to this article as no data sets were generated or analyzed during this study.
